# Identification of people with Lynch syndrome from those presenting with colorectal cancer in England: baseline analysis of the diagnostic pathway

**DOI:** 10.1038/s41431-024-01550-w

**Published:** 2024-02-15

**Authors:** Fiona E. McRonald, Joanna Pethick, Francesco Santaniello, Brian Shand, Adele Tyson, Oliver Tulloch, Shilpi Goel, Margreet Lüchtenborg, Gillian M. Borthwick, Clare Turnbull, Adam C. Shaw, Kevin J. Monahan, Ian M. Frayling, Steven Hardy, John Burn

**Affiliations:** 1 National Disease Registration Service, NHS England, London, UK; 2 Health Data Insight, Cambridge, UK; 3 Guy’s and St. Thomas’ NHS Foundation Trust, London, UK; 4 Cancer Epidemiology and Cancer Services Research, King’s College London, London, UK; 5 Translational and Clinical Research Institute, Newcastle University, Newcastle upon Tyne, UK; 6 The Institute of Cancer Research, Sutton, UK; 7 St Mark’s Hospital Centre for Familial Intestinal Cancer, Imperial College, London, UK; 8 St Vincent’s University Hospital, Dublin, Ireland

**Keywords:** Cancer genetics, Colorectal cancer, Genetic testing, Diagnostic markers, Cancer screening

## Abstract

It is believed that >95% of people with Lynch syndrome (LS) remain undiagnosed. Within the National Health Service (NHS) in England, formal guidelines issued in 2017 state that all colorectal cancers (CRC) should be tested for DNA Mismatch Repair deficiency (dMMR). We used a comprehensive population-level national dataset to analyse implementation of the agreed diagnostic pathway at a baseline point 2 years post-publication of official guidelines. Using real-world data collected and curated by the National Cancer Registration and Analysis Service (NCRAS), we retrospectively followed up all people diagnosed with CRC in England in 2019. Nationwide laboratory diagnostic data incorporated somatic (tumour) testing for dMMR (via immunohistochemistry or microsatellite instability), somatic testing for *MLH1* promoter methylation and *BRAF* status, and constitutional (germline) testing of MMR genes. Only 44% of CRCs were screened for dMMR; these figures varied over four-fold with respect to geography. Of those CRCs identified as dMMR, only 51% underwent subsequent diagnostic testing. Overall, only 1.3% of patients with colorectal cancer had a germline MMR genetic test performed; up to 37% of these tests occurred outside of NICE guidelines. The low rates of molecular diagnostic testing in CRC support the premise that Lynch syndrome is underdiagnosed, with significant attrition at all stages of the testing pathway. Applying our methodology to subsequent years’ data will allow ongoing monitoring and analysis of the impact of recent investment. If the diagnostic guidelines were fully implemented, we estimate that up to 700 additional people with LS could be identified each year.

## Introduction

At least 3% of cancers are attributable to constitutional (germline) pathogenic variants in a cancer susceptibility gene (CSG) [1]. Families harbouring these constitutional pathogenic variants were classically ascertained by clinical geneticists, based on familial clustering of related tumour types in several relatives, multiple primary tumours in some individuals, and tumour development at a younger age than typical for that cancer type. However, more widespread availability of molecular diagnostics has revealed other individuals who carry a similar genetic predisposition, but with a more subtle familial phenotype, or absence of a family history of similar cancers [2, 3]. Ascertainment has therefore been biased towards the classical familial pattern rather than the individual’s own phenotype.

The Mismatch Repair (MMR) family of proteins is responsible for rectifying DNA replication errors that arise during the S-phase of the cell cycle. Germline pathogenic variants affecting any of the four MMR genes *MLH1*, *MSH2*, *MSH6* or *PMS2* underlie Lynch syndrome (LS), conferring a strong predisposition towards various cancers—predominantly colorectal and endometrial carcinoma, but also others including urothelial, ovarian, and upper gastrointestinal cancers, and sebaceous dermatological tumours [4]. Estimates of the true population prevalence of LS [5–7] indicate substantial underdiagnosis, hence NHS England’s imperative to identify more cases. Outcomes for people diagnosed with LS could be improved by offering regular colonoscopy, aspirin and prophylactic gynaecological surgery, leading to reduced cancer incidence and earlier diagnosis. This could result in significant financial savings across the NHS [8], in addition to the primary objective of saving lives.

National Institute for Health and Care Excellence (NICE) guidelines (DG27) [9] issued in February 2017 state that all colorectal cancers (CRC) should be tested for MMR deficiency (dMMR) at the point of diagnosis, using either immunohistochemistry (IHC) or microsatellite instability (MSI) testing. Any tumours with evidence of dMMR should undergo further molecular tests, culminating in germline MMR gene testing for individuals at highest likelihood of having LS. In 2018, the charity Bowel Cancer UK initiated a Freedom of Information request [10] and campaign [11]—‘Time To Test’—finding that MMR testing guidelines were being implemented by only 17% of hospitals in England, with cited barriers to testing including funding, staff capacity, awareness and local policy.

Whilst the diagnostic guidelines are clear, it is important to evaluate whether these are being consistently applied across the different NHS Cancer Alliances (regional healthcare partnerships that drive integration of local cancer services), and to highlight any inequities. This requires large scale, population-level collection and curation of molecular testing data, and robust linkage to cancer diagnoses. The National Disease Registration Service (NDRS) has developed a programme of work collating germline and somatic genetic testing data from NHS laboratories. By linking these data at patient- and tumour-level to national cancer registration records [12], we are, for the first time, able to describe the English national landscape of LS molecular diagnostic testing. The baseline data presented here refer to all colorectal cancers diagnosed in England in the year 2019, the first year for which national molecular data collections made this possible.

## Methods

### Cancer registration

The National Cancer Registration and Analysis Service (NCRAS), part of NHS England, constructs the population-based cancer registry for England [12]. Somatic genomic testing data was derived from two sources: bespoke data extracts supplied by individual genomic laboratories, and pathology reports acquired through the nationally mandated Cancer Outcomes and Services Dataset (COSD). Laboratory germline data on MMR genes was submitted and processed via pseudonymisation and bioinformatics pipelines previously described [13], and linked at patient-level. Somatic data was linked at tumour-level. Where MMR testing was referenced in the initial pathology report, but there was no supplementary report containing the MMR test results, this was fed back to the relevant NHS Trust by the NCRAS Data Improvement Team, to maximise national data completeness.

### Data analysis

From the 2019 end of year cancer registration table, 37,662 colorectal tumours (10th revision of the International Classification of Diseases (ICD-10) C18, C19 or C20) diagnosed in 2019 were identified. All tumours were linked to the genomic testing data up to the end of 2020 (latest available data at the time of writing).

From the cancer registry data, information on patients’ demographics and tumour information was retrieved. Patients were assigned a Cancer Alliance based upon their postcode of residence at diagnosis, using the 2019 geographical boundaries. Age groups were banded from 10–29 years, then by 10-year intervals between 30–49 years, 5-year intervals between 50–89 years, then 90 years+.

Self-reported gender and ethnicity information is recorded in the cancer registration data from clinical records; ethnicity was categorised according to the 16-category classification as used in the 2021 Census of England and Wales. This was then collapsed to seven ethnic groups: White, Asian, Chinese, Black, Mixed, Other, and Unknown. Each patient’s socioeconomic deprivation quintile was assigned using the patient’s residential postcode at the time of diagnosis and based upon the quintile distribution of the lower-layer super output area (LSOA) ranking of the Indices of Multiple Deprivation (IMD) 2019, with 1 being the most deprived and 5 being the least deprived. Tumour stage is recorded according to the Union for International Cancer Control (UICC) Classification of Malignant Tumours (TNM). Colorectal cancer grading is recorded as 1 to 4, with 1 representing well differentiated cancer cells through to 4 when cancer cells are poorly differentiated or undifferentiated.

Descriptive statistics, chi-squared and *t*-tests, and logistic regression analyses were carried out using R software [14].

### Ethical and legal considerations

The data included in this study were collected and analysed under the National Disease Registries Directions 2021 [15], made in accordance with sections 254(1) and 254(6) of the 2012 Health and Social Care Act.

Before embarking upon the collection of genetic data, we sought courtesy permission from the Caldicott Guardian at each NHS Trust housing the relevant laboratories.

### Patient and public involvement

Author JB has been involved with the patient group Lynch Syndrome UK (LSUK) from when it was established as a charity in 2014, initially as the Clinical Director. JB, GMB, FEM, IMF and KJM have all presented work in progress to LSUK at their annual conference, and are in regular contact, receiving patient feedback.

## Results

### Somatic testing

In 2019, 37,662 CRCs (from 37,090 people) were diagnosed in England. Under half of these (44%; 16,463) were tested for dMMR. IHC was the preferred test method in 89% of cases; the remainder were tested by MSI (8%) or by both methods (3%). The dMMR detection rates were slightly higher for MSI (19% detection rate) than for IHC (16% detection rate) (*χ*^2^ = 12.0; df = 1; *p* < 0.01).

To triage individuals for germline testing as per NICE guidelines, dMMR tumours can be further subdivided according to MLH1 status. Individuals whose tumours are proficient for MLH1, but abnormal for one or more of the other MMR proteins (MSH2, MSH6 or PMS2), should be offered direct referral for germline testing; tumours with MLH1 abnormality require further somatic tests.

Overall, 16% (*n* = 2576) of CRCs were dMMR. Of these, 15% (*n* = 386 tumours from 372 patients) were deficient in MSH2, MSH6 or PMS2 (but MLH1-proficient), so were eligible for germline testing; 121 of these patients (33%) received a germline test. The remaining 85% (*n* = 2190) CRCs were MLH1-deficient or MSI-High, indicating requirement for further somatic tests. Downstream testing was, however, performed on only 54% (*n* = 1178) of these, comprising 1041 tumours tested for *BRAF* mutational status and a further 137 tested for *MLH1* promoter hypermethylation in the absence of *BRAF* testing.

**Fig. 1:**
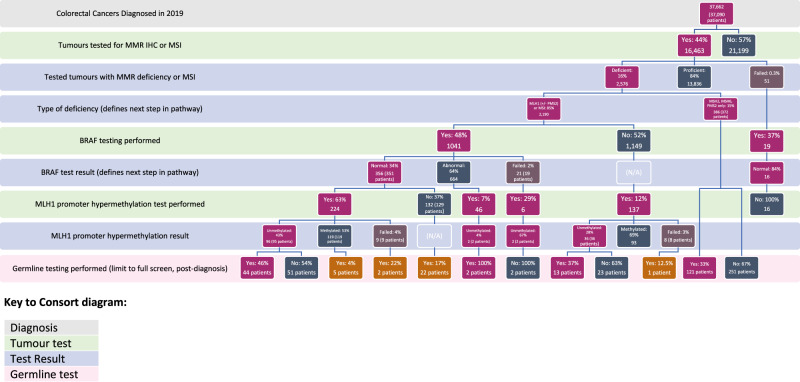
Consort diagram showing Lynch syndrome testing pathway from cancer diagnosis to germline testing in 37,662 colorectal cancers (from 37,090 patients) diagnosed in England in 2019. For all levels of the Consort diagram, borderline results have been categorised as eligible to proceed to the next stage of the testing pathway, e.g. ‘deficient’ box in ‘tested tumours with MMR deficiency or MSI’ row includes both abnormal and borderline results; ‘proficient’ box includes normal results only; ‘failed’ box includes everything else (failed/not tested/unknown). Dark pink boxes represent the NICE DG27 ‘official’ pathway to germline testing, defined as MMR deficiency with (in the case of MLH1 deficiency or MSI-High status), an unmethylated *MLH1* promoter. An unbroken line of pink boxes from top to bottom indicates the ‘textbook’ NICE-recommended pathway. Other pink boxes show paths to germline testing performed on samples that were incompletely tested, but were MLH1 deficient and unmethylated. Orange boxes indicate germline tests done under broader inclusion criteria, i.e. MLH1 deficiency with *BRAF* wild type but *MLH1* promoter methylated, failed testing, or untested. Dark grey boxes indicate either a lack of testing, or a test result that would signify a legitimate end to the testing pathway. Light brown boxes indicate failed tests.

Of those MLH1-deficient CRCs tested for *BRAF*, 34% (*n* = 356) had a normal (i.e. wild-type) result, of which 63% (*n* = 224) were reflex tested for *MLH1* promoter hypermethylation, as per NICE guidance. An additional 52 *MLH1* promoter tests were performed following abnormal or failed *BRAF* results. Thus a total of 413 tumours were tested for *MLH1* promoter hypermethylation as part of the Lynch screening pathway, of which 138 tumours (33%), from 137 patients, were unmethylated, and therefore eligible for germline testing. Full testing pathways and results are shown in Fig. 1.

### Variation in MMR testing

**Fig. 2:**
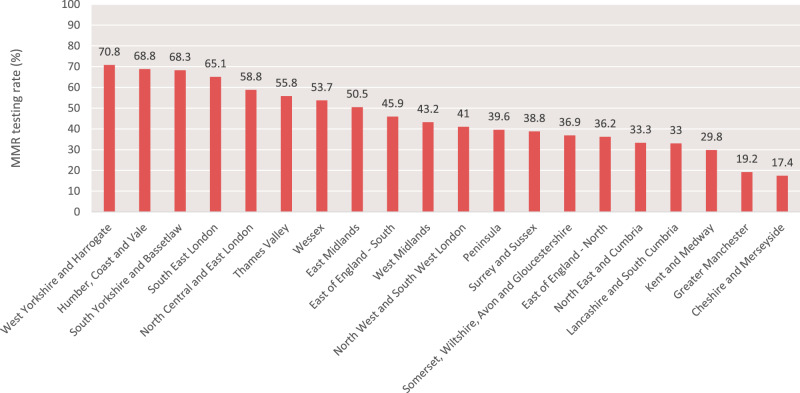
Geographical variation in compliance with guidelines to test all CRCs for dMMR. Proportion of 2019-diagnosed colorectal cancers tested for dMMR, stratified by NHS England Cancer Alliance (using 2019 geographical boundaries and based upon patient postcode of residence at diagnosis).

Table 1 shows numbers and percentages of people having MMR testing according to patient and tumour characteristics. Females had a slightly lower testing rate than males (42.8% vs. 44.5%). The lowest testing rates were found among persons of White (43.4%) or unknown (39.8%) ethnicity, whereas the highest testing rate was observed among Black persons (56.5%). Testing rates were highest among persons from the least deprived areas (45.8%) and lowest among those from the most deprived areas (40.8%). Higher testing rates were observed for tumours with stage II and III (52.1% and 52.6%, respectively) than for stage I (40%) and IV (41%) and tumours with unknown stage (27.9%). Similarly, higher testing rates were found among grade 2 (52.8%) and 3 (53.7%) tumours than grade 1, 4 and unknown grade tumours (32.8%, 27.5% and 15.8%, respectively). The most striking difference in MMR testing rates was according to Cancer Alliance, where tumour MMR testing rates varied from 17 to 71% (Fig. 2). When compared to the Cancer Alliance with the highest testing rate (West Yorkshire and Harrogate), and apart from the surrounding Cancer Alliances (Humber, Coast and Vale, and South Yorkshire and Bassetlaw), tumours diagnosed in all other Cancer Alliances were significantly less likely to be tested; more markedly so when adjusting for demographic differences between Cancer Alliances. Full outcomes from the uni- and multivariable logistic regression analyses are shown in Supplementary Table 1.

**Table 1:** MMR testing according to patient and tumour characteristics.

	MMR tested?				
	No (*n* | %)		Yes (*n* | %)		*χ*^2^ *p* value
Total	21,199	56.3%	16,463	43.7%	
Age					<0.001
10–29	40	38.1%	65	61.9%	
30–39	228	31.1%	504	68.9%	
40–49	450	30.9%	1006	69.1%	
50–54	675	40.4%	995	59.6%	
55–59	1289	47.2%	1444	52.8%	
60–64	1965	49.7%	1986	50.3%	
65–69	2239	50.4%	2203	49.6%	
70–74	3472	54.2%	2935	45.8%	
75–79	3196	57.4%	2374	42.6%	
80–84	3501	64.8%	1904	35.2%	
85–89	2670	76.0%	842	24.0%	
90+	1474	87.8%	205	12.2%	
Gender					0.001
Female	9581	57.2%	7161	42.8%	
Male	11,618	55.5%	9302	44.5%	
Ethnicity					<0.001
Asian	363	43.8%	466	56.2%	
Black	261	43.5%	339	56.5%	
Chinese	46	48.9%	48	51.1%	
Mixed	79	52.3%	72	47.7%	
Other	250	52.1%	230	47.9%	
Unknown	1689	60.2%	1117	39.8%	
White	18,511	56.6%	14,191	43.4%	
Socioeconomic deprivation quintile					<0.001
1—Most deprived	3571	59.2%	2463	40.8%	
2	3753	55.6%	3000	44.4%	
3	4584	57.3%	3410	42.7%	
4	4720	55.9%	3720	44.1%	
5—Least deprived	4571	54.2%	3870	45.8%	
Cancer alliance					<0.001
Cheshire and Merseyside	1497	82.6%	315	17.4%	
East Midlands	1691	49.5%	1724	50.5%	
East of England—North	1399	63.8%	793	36.2%	
East of England—South	1255	54.1%	1065	45.9%	
Greater Manchester	1424	80.8%	338	19.2%	
Humber, Coast and Vale	332	31.2%	731	68.8%	
Kent and Medway	874	70.2%	371	29.8%	
Lancashire and South Cumbria	883	67.0%	435	33.0%	
North Central and East London	580	41.2%	829	58.8%	
North East and Cumbria	1561	66.7%	780	33.3%	
North West and South West London	1015	59.0%	706	41.0%	
Peninsula	877	60.4%	576	39.6%	
Somerset, Wiltshire, Avon and Gloucestershire	1353	63.1%	792	36.9%	
South East London	304	34.9%	566	65.1%	
South Yorkshire and Bassetlaw	324	31.7%	698	68.3%	
Surrey and Sussex	1423	61.2%	904	38.8%	
Thames Valley	688	44.2%	870	55.8%	
Wessex	877	46.3%	1016	53.7%	
West Midlands	2366	56.8%	1798	43.2%	
West Yorkshire and Harrogate	476	29.2%	1156	70.8%	
Tumour stage					<0.001
I	3614	60.0%	2413	40.0%	
II	3740	47.9%	4076	52.1%	
III	4555	47.4%	5045	52.6%	
IV	4314	59.0%	3000	41.0%	
Unknown	4976	72.1%	1929	27.9%	
Tumour grade					<0.001
1	868	67.2%	424	32.8%	
2	10,842	47.2%	12,117	52.8%	
3	2200	46.3%	2550	53.7%	
4	29	72.5%	11	27.5%	
Unknown	7260	84.2%	1361	15.8%	

Data shown as absolute numbers and proportions.

### Access to somatic follow up testing

Significant variation between Cancer Alliances was also observed when considering follow up of dMMR tumours (either germline testing for MSH2/MSH6/PMS2 deficient tumours, or further somatic testing for MLH1 deficient tumours). Performance of Cancer Alliances on follow up metrics did not necessarily correspond to their performance in arranging initial MMR testing (Supplementary Fig. 1).

### Constitutional (germline) testing

Overall, 507 individuals with CRC were eligible for germline testing based on NICE guidelines—i.e. their tumours were either abnormal for MSH2/MSH6/PMS2 (*n* = 372) or abnormal for MLH1/MSI-High with no evidence of *MLH1* promoter methylation (*n* = 135). Of these 507 people, just 36% (*n* = 180) received a germline full screen test following their diagnosis. If eligibility for germline testing is instead based upon the NHS National Genomic Test Directory (indication R210) [16], this includes all patients whose MLH1-deficient/MSI-High tumours are *BRAF* wild-type (i.e. skipping *MLH1* promoter methylation testing). Adopting these broader eligibility criteria—i.e. at least one of *BRAF* wild type or failed, or *MLH1* promoter unmethylated or failed—786 people with CRC could have been offered a germline test. Of these 786 patients, 36 (5%) had either already received a germline test before diagnosis, or received a targeted germline test after diagnosis—i.e. they were members of families already known to genetics services. Thus 750 patients were, as a result of tumour molecular testing, newly identified as being eligible for germline testing, of whom only 210 (28%) actually received a germline test.

Of all 37,090 patients diagnosed with CRC in 2019, 487 (1.3%) received germline MMR testing (Table 2). Those tested could be split into four groups, depending on (1) the timing of the germline test with respect to the 2019 CRC diagnosis (pre- or post-diagnosis), and (2) the scope of the germline test (full screening of all MMR genes, versus targeted testing for a specific pathogenic variant in a member of a known LS family) (Table 2). This distinction is important, as it reflects how patients were ascertained, and thus what proportion were identified through the NICE-recommended tumour testing pathway, as opposed to being already known to clinical genetics services.

**Table 2:** Number of germline MMR tests performed in 2019, split by test timing and scope.

		Timing of germline test, with respect to CRC diagnosis in 2019		
		Pre-diagnosis germline test	Post-diagnosis germline test	Total
Scope of germline test	Full screen test (Interrogates all MMR genes for an unknown variant)	41	390	431
	Targeted test (Looks for a specific MMR gene variant already known to segregate in family members)	30	26	56
	Total	71	416	487

A minority of germline tests (56/487; 11%) were targeted tests; these are indicated when a specific pathogenic variant has previously been identified in a relative. Of these, germline testing preceded the 2019 CRC diagnosis (i.e. predictive/pre-symptomatic testing) in 30 (54%); the remaining 26 (46%) underwent confirmatory germline testing following their CRC diagnosis.

Forty-one people (8% of all tested) had full screen testing prior to their 2019 CRC diagnosis; this could either follow an earlier cancer diagnosis, or be a clinical genetics referral for ‘indirect testing’ where family history or personal polyp status was sufficiently strong to warrant variant-agnostic germline testing.

Three hundred and ninety out of 487 germline tests (80%) were full screen, post-diagnosis tests; this group represents newly-identified LS families, as opposed to those already known to genetics services. However, not all 390 tests were performed as per NICE or National Genomic Test Directory guidelines (Table 3). Even taking the more liberal eligibility criteria for germline testing, as outlined above [16], only 210 out of 390 (54%) followed recommended diagnostic pathways. The remainder comprised 45 people whose tumour records showed no evidence of dMMR testing, 74 with MMR proficient tumours, 53 with MLH1 deficiency/MSI-High status but no evidence of downstream somatic testing, and eight with MLH1 deficiency but mutant *BRAF*/*MLH1* promoter hypermethylation.

**Table 3:**
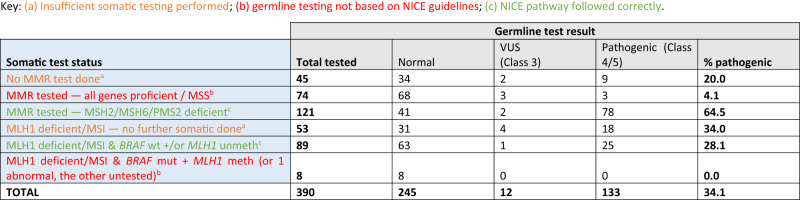
Full screen, post-diagnosis germline tests, split by route to testing (somatic test status), and outcome of the germline test.

Thus of the total 390 full screen, post-diagnosis germline tests carried out, 210 patients (54%) were tested appropriately, 98 (25%) with no or insufficient somatic testing, and 82 (21%) following somatic results that did not indicate germline testing.

### Overlap between somatic and germline testing

**Fig. 3:**
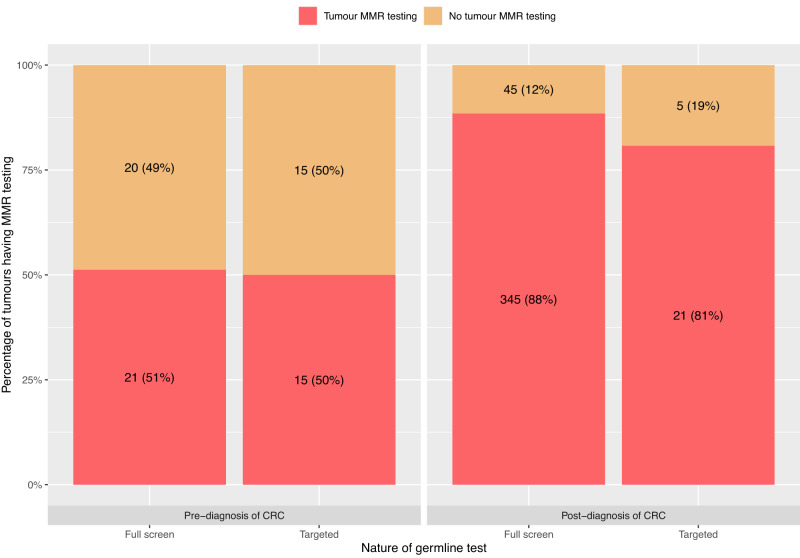
Number and percent of tumours having dMMR testing, grouped by timing of patient’s germline genetic test (pre- or post-2019 diagnosis of CRC) and scope of their germline test (full screen or targeted). Bars from L to R: full screen germline test performed pre-2019 cancer diagnosis; targeted germline test performed pre-2019 cancer diagnosis; full screen germline test performed post-2019 cancer diagnosis; targeted germline test performed post-2019 cancer diagnosis. Red bars signify that tumour dMMR testing has taken place; orange bars indicate no tumour dMMR test was performed.

Individuals having a germline test post-diagnosis were significantly more likely (*χ*^2^ = 58; *p* < 0.0001) to have had MMR testing on their 2019-diagnosed tumour(s) (366/416; 88%) than those whose germline test had preceded their 2019 CRC diagnosis (36/71; 51%). The group most likely to have had MMR tumour testing were the full screen, post-diagnosis germline test group, at 88%) (Fig. 3).

### Outcome of germline testing

A germline MMR pathogenic or likely pathogenic (P/LP) variant was reported in 206/487 (42%) people tested, comprising variants detected in 156/431 (36%) people undergoing full screen testing, and 50/56 (89%) people undergoing targeted (familial) testing. Abnormal germline results were distributed between the four MMR genes and *EPCAM* as expected [4], with variants in *MLH1* and *MSH2* comprising 65% of cases, and *PMS2* just 15% (Table 4).

**Table 4:** Mutated gene spectrum for all 2019-diagnosed colorectal cancer patients who had an abnormal germline Lynch test (*n* = 206; includes all germline test scopes and timings).

Gene	Number of patients with pathogenic/likely pathogenic variant, split by gene (*N* = 206)	Proportional distribution by gene of all patients with pathogenic/likely pathogenic variant (%)
*MLH1*	65	31.6
*MSH2*	68	33.0
*MSH6*	41	19.9
*PMS2*	31	15.0
*EPCAM*	1	0.5

When full screen, post-diagnosis germline tests were stratified according to prior somatic testing status, variant detection rates ranged from 0–64%, (Table 3). A P/LP variant was detected in 103/210 (49%) of people whose tumour testing pathway followed NICE guidelines, in 27/98 (28%) of those where somatic testing was absent or incomplete, and in 3/82 (4%) of those where somatic testing results did not indicate germline testing (Table 3).

Of patients undergoing full screen, post-diagnosis germline testing, those with MMR-proficient (pMMR) tumours were significantly younger than those with dMMR tumours (mean 48.1 years vs. 56.8 years; Welch two sample *t-*test statistic = 4.29 (95% CI = 4.67–12.68, df = 111.78, *p* = 0.001).

### Timeline of complete molecular diagnostic pathway for LS

**Fig. 4:**
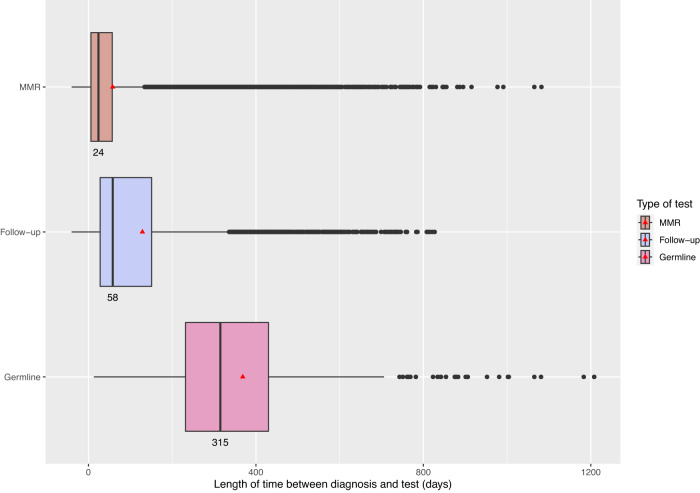
Distribution and average time from initial diagnosis (at day 0) to functional testing (MMR IHC/MSI), subsequent follow-up (somatic *BRAF*/*MLH1* promoter methylation testing following an MMR test) and germline testing. Within each box, vertical black lines denote median values (enumerated below the box), and red triangles denote mean values; boxes extend from the 25th to the 75th percentile of each group’s distribution of values and denote the interquartile range (IQR). Horizontal extending black lines denote adjacent values (i.e. the most extreme values within 1.5 x IQR of the 25th and 75th percentile of each group); black dots denote the observations outside the range of adjacent values (i.e. the outliers). Only full screen, post-diagnosis germline tests are included here (pre-diagnosis tests went back ~18 years).

The median time between CRC diagnosis and functional MMR testing (IHC and/or MSI) was 24 days (mean 58 days), with a further 34 days elapsing before follow up somatic testing, i.e. the total median time to complete somatic testing was 58 days (mean 129 days). The main diagnostic pathway delay occurred between somatic and germline testing, the latter being performed at median 315 days (mean 368 days) following initial CRC diagnosis. For all tests, there was a long right-hand tail in the distribution, indicating delays exceeding 1000 days for some individuals (Fig. 4).

## Discussion

This is the first comprehensive analysis of a policy to identify people with Lynch syndrome (LS) across a national healthcare system serving 55 million people. Despite being a snapshot in time, prior to coordinated expansion of testing [17], it provides a baseline for assessment of future developments, and is a likely reflection of underdiagnosis of this treatable disorder in other developed countries [18, 19].

In depth analysis of comparable populations suggests a LS birth prevalence of 1 in 280–1 in 500 [5–7], implying a population prevalence of one to two hundred thousand in England. Pooled data across clinical and laboratory genetics services indicates under 10% are known. A health economic analysis [8] indicated the clinical utility of testing all CRCs for dMMR; on this evidence, NICE introduced the current pathway in 2017 [9]. The rationale for identifying LS carriers is further enhanced by the demonstration of a 50% reduction in their CRC incidence following daily aspirin [20] (now also a NICE guideline) [21], and the highly significant reduction in their non-CRC LS-associated cancer risk when prescribed dietary supplementation with resistant starch [22]. Identification of dMMR cancers as a target for immunotherapy [23–26] provides further justification for functional testing of all tumours, regardless of patient LS status.

The health economic benefit can be maximised by offering cascade testing to relatives to identify other at-risk carriers. Management guidelines for LS are gene-specific: colonoscopic surveillance should be offered at least every 2 years, starting from age 25 for carriers of pathogenic or likely pathogenic variants (PVs) in *MLH1* or *MSH2*, and from age 35 for those with PVs in *MSH6* or *PMS2* [27]. From 2023, colonoscopic surveillance of LS carriers will be incorporated into the NHS national bowel cancer screening programme.

Any guidelines, however good, are only beneficial if properly implemented. The Bowel Cancer UK investigation in 2018 indicated that only 17% of hospitals in England were following NICE recommendations for tumour MMR testing [10]; however this questionnaire-based investigation was limited in its design, potentially had a response-bias, and was set up to ask the question at hospital-level rather than patient-level. The current study is therefore the first national evaluation of MMR testing in England, covering the entire LS diagnostic pathway from initial tumour testing (IHC/MSI) through to germline testing, and is only possible due to the systematic collection, curation, and linkage of comprehensive NHS laboratory data within the National Disease Registration Service (NDRS).

Our data show that only 44% of 2019-diagnosed CRCs were tested for MMR status (IHC and/or MSI), and highlight large disparities in provision across England. There was more than a four-fold difference in MMR testing rates between the best- and worst-performing Cancer Alliance. Notably, the three best performing Cancer Alliances (West Yorkshire and Harrogate, South Yorkshire and Bassetlaw, and Humber, Coast and Vale) belong to the Yorkshire and Humber (YH) region, where, between April 2017 and March 2019, the Yorkshire Cancer Research Bowel Cancer Improvement Programme (YCR BCIP) funded pilot MMR screening for all CRC patients in the region who were not already covered by the previous inclusion criteria (<50 years of age) [28]. Although the YH pilot overlapped this NDRS evaluation only for the first 3 months of 2019, the region performed consistently well throughout the year, indicating the ongoing positive legacy of the YCR BCIP programme, and its implementation of suitable infrastructure, education, and co-ordination.

Of the 16% of tested tumours found to be dMMR, only 51% were followed up as per diagnostic guidance: 121/372 (33%) patients with MSH2/MSH6/PMS2 deficient tumours had germline testing, and 1178/2190 (54%) tumours with MLH1 deficiency or MSI-High status had further somatic testing. The latter facilitates distinction between sporadic (tumour-confined) dMMR versus potential constitutional dMMR underpinned by a germline pathogenic variant. As with initial MMR testing, the follow up of dMMR tumours was observed to vary significantly across Cancer Alliances.

There are some caveats here around data completeness, with potential gaps in *BRAF* data particularly affecting London and the Thames Valley region. Additionally, due to database challenges at genomics laboratories, we are missing a small number of germline MMR testing records from Great Ormond Street from December 2019 onwards, and from Bristol since the inception of their MMR testing service in summer 2019. Nevertheless, these gaps constitute a very small proportion of the overall national LS-related testing activity, and do not alter our overall conclusions. In 2019, 2 years after publication of the NICE guidance [9], MMR testing and appropriate follow up were generally poorly implemented, with major geographical inequities, substantial attrition from all levels of the testing pipeline, and very long time lags between initial functional MMR tumour testing and germline follow up. This long delay in germline testing limits the analysis that can be performed on more recently diagnosed tumours, as the data need time to mature with respect to the time period between diagnosis of cancer and genetic diagnosis of Lynch syndrome. It also evidences the need to develop and implement more efficient LS testing pathways, e.g. those co-ordinated via mainstream oncology services.

Where germline testing was performed, we observed a relatively high detection rate of pathogenic/likely pathogenic (P/LP) variants. Amongst full screen, post-diagnosis tests, the detection rate was 34.1%; this is somewhat higher than the 28% reported for all full screen MMR testing carried out in English labs since 2008 [13]. The difference probably reflects the biased nature of the 2019 CRC-diagnosed cohort, most of whose tumours had been pre-screened for dMMR. In contrast, most historical full screen germline testing would have been performed based on family history and/or young age of cancer development. Accordingly, by restricting the 2019 analysis to patients whose tumour-screening adhered properly to the NICE guidelines, the germline detection rate increased to 49%. Strikingly, a germline P/LP MMR variant was detected in 65% of patients whose tumours were abnormal for MSH2/MSH6/PMS2, indicating the clinical utility of this as a biomarker of LS.

Overall, 133 (65%) of the total 206 people with MMR germline P/LP variants were identified following a full screen, post-diagnosis germline test, i.e. represented new LS families not previously known to genetics services. This demonstrates the importance of the NICE-recommended tumour testing pathway in identifying new cases. Were the pathways to be implemented fully, both lives and health service resources could be saved [8, 29]. Based on extrapolations from all tumour and germline data, we estimate that, were NICE guidelines to be fully executed in all cases of CRC, up to 700 additional LS index cases (above this 2019 baseline) could be diagnosed per year; others could then be identified through familial testing.

Since the current reporting period of 2019 diagnoses, there has been more recognition of the importance of detecting LS, and a national transformation project is now underway [17]. This report provides a baseline for the anticipated improvement in LS detection. To facilitate comparison, and provide figures for subsequent reporting years beyond this baseline, we have made regional and national data available online at https://cancerstats.ndrs.nhs.uk/molecular/lynchsyndrome (requires an NHS network connection and login).

The national-scale collection, collation, curation and standardisation of these data by NDRS is the world’s first example of linking cancer records with both germline and somatic molecular testing data in a real-world setting at population-level. Linkage of genomic data to the rich clinical phenotype, treatment and outcome data held within NDRS will enable the NHS to build up a comprehensive picture of genotype-phenotype correlations, facilitate genetic counselling of families with cancer, and monitor equity of access to molecular testing and targeted therapies. Through our collaboration with the UK Cancer Variant Interpretation Group (CanVIG-UK) [30], the datasets are also supporting national efforts to interpret germline variants of uncertain clinical significance (VUS).

## Conclusion

The data presented here for 2019 diagnoses of colorectal cancer are the first of their kind to give a national picture of Lynch syndrome diagnostics across the entire cancer pathway, encompassing both germline and somatic testing. Only 44% of CRCs were screened for MMR deficiency; these figures varied over four-fold with respect to geography. These 2019 figures provide a baseline level of tumour testing and indicate the level of underdiagnosis of LS at a point 2 years from when NICE recommended MMR testing in all colorectal cancers, but prior to the widespread disruption to NHS services caused by the SARS-CoV-2 pandemic. Now that the national data collection, processing, and analytical methodology is embedded within NDRS, it is possible to monitor improvements over time, and to benchmark the relative performance of individual NHS Trusts and Cancer Alliances.

### Supplementary information

Supplementary Figure 1

Supplementary Table 1

Legends for Supplementary Material

## Data Availability

Data are held within the National Disease Registration Service (NDRS), which is part of NHS England. Formal data requests may be made through the Data Access Request Service (DARS): https://digital.nhs.uk/services/data-access-request-service-dars.

## Code availability

Analytical code is available from the National Disease Registration Service (NDRS) upon reasonable request.
