# Supplementary figures and images for: Identification of people with Lynch syndrome from those presenting with colorectal cancer in England: baseline analysis of the diagnostic pathway
Source: Eur J Hum Genet. 2024 Feb 15;32(5):529–38. doi: 10.1038/s41431-024-01550-w (PMC11061113; doi:10.1038/s41431-024-01550-w)

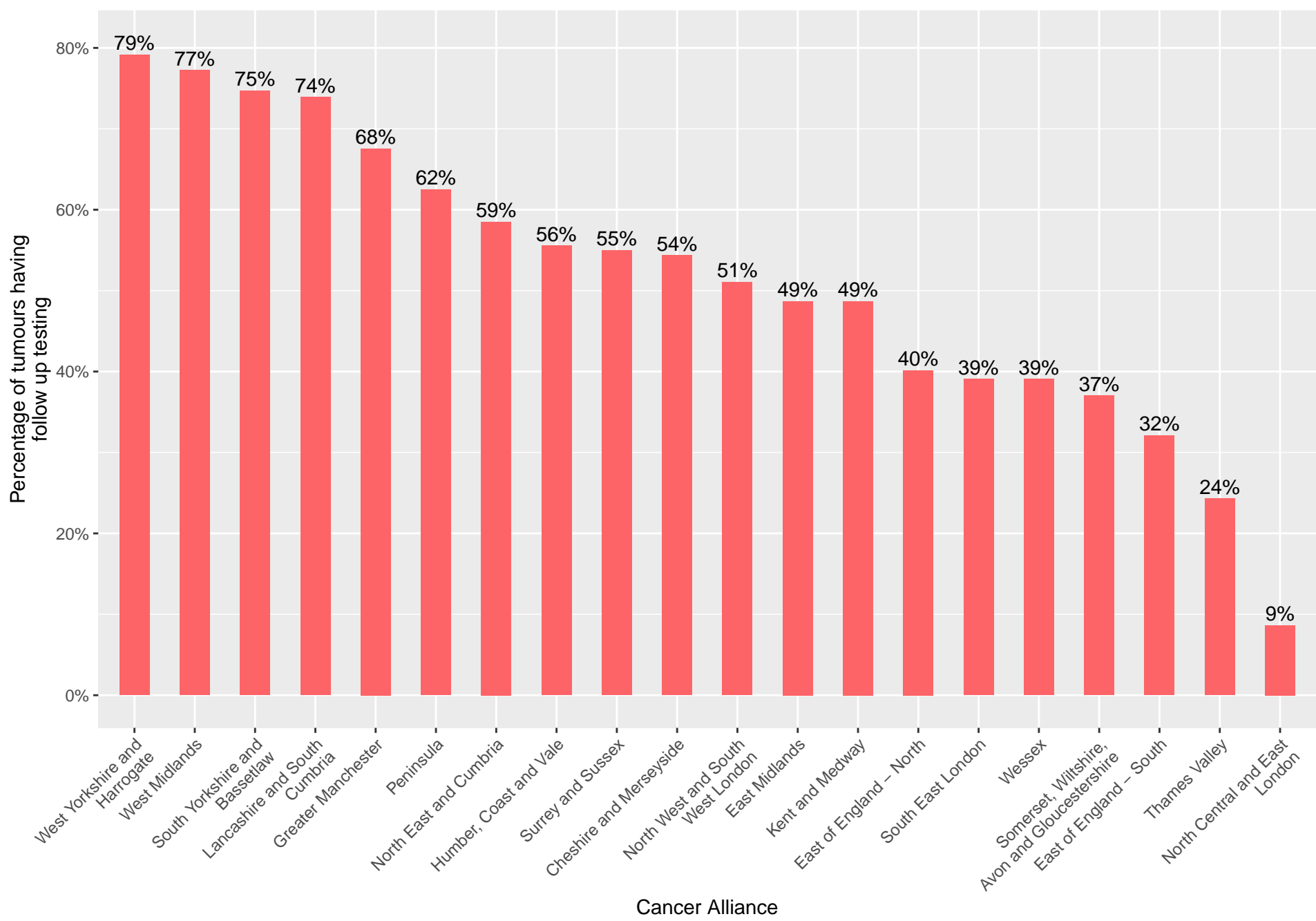

Supplement: Supplementary file 1 — Supplementary Figure 1 [file 41431_2024_1550_MOESM1_ESM.pdf]
