# Supplementary material for: Identification of people with Lynch syndrome from those presenting with colorectal cancer in England: baseline analysis of the diagnostic pathway
Source: Eur J Hum Genet. 2024 Feb 15;32(5):529–38. doi: 10.1038/s41431-024-01550-w (PMC11061113; doi:10.1038/s41431-024-01550-w)
Supplement: Supplementary file 2 — Supplementary Table 1 [file 41431_2024_1550_MOESM2_ESM.docx]

**Supplementary Table 1:** Likelihood of colorectal cancer patients receiving mismatch repair (MMR) testing through immunohistochemistry (IHC) or microsatellite instability (MSI) methods in England in 2019, according to patient and tumour characteristics and Cancer Alliance at diagnosis. Univariable and multivariable logistic regression modelling.

|  | | **Univariable** | | |  | **Multivariable** | | |
| --- | --- | --- | --- | --- | --- | --- | --- | --- |
|  | | **OR^a^** | **95% CI** | |  | **OR^b^** | **95% CI** | |
| **Patient characteristics** | |  |  |  |  |  |  |  |
| **Age** | |  |  |  |  |  |  |  |
| 10-29 | | 1.92 | 1.30 | 2.88 |  | 1.87 | 1.23 | 2.85 |
| 30-39 | | 2.61 | 2.22 | 3.09 |  | 2.81 | 2.37 | 3.34 |
| 40-49 | | 2.64 | 2.34 | 2.99 |  | 2.81 | 2.47 | 3.19 |
| 50-54 | | 1.74 | 1.56 | 1.95 |  | 1.79 | 1.60 | 2.01 |
| 55-59 | | 1.33 | 1.21 | 1.45 |  | 1.37 | 1.25 | 1.51 |
| 60-64 | | 1.20 | 1.10 | 1.29 |  | 1.22 | 1.12 | 1.33 |
| 65-69 | | 1.16 | 1.08 | 1.26 |  | 1.20 | 1.11 | 1.30 |
| 70-74 | | 1.00 | (reference) | |  | 1.00 | (reference) | |
| 75-79 | | 0.88 | 0.82 | 0.94 |  | 0.88 | 0.81 | 0.95 |
| 80-84 | | 0.64 | 0.60 | 0.69 |  | 0.62 | 0.57 | 0.67 |
| 85-89 | | 0.37 | 0.34 | 0.41 |  | 0.34 | 0.31 | 0.38 |
| 90+ | | 0.16 | 0.14 | 0.19 |  | 0.14 | 0.12 | 0.17 |
| p-trend | | *<0.001* |  |  |  | *<0.001* |  |  |
| **Gender** | |  |  |  |  |  |  |  |
| Male | | 1.00 | (reference) | |  | 1.00 | (reference) | |
| Female | | 0.93 | 0.90 | 0.97 |  | 0.99 | 0.95 | 1.03 |
| **Ethnicity** | |  |  |  |  |  |  |  |
| White | | 1.00 | (reference) | |  | 1.00 | (reference) | |
| Asian | | 1.67 | 1.46 | 1.92 |  | 1.23 | 1.06 | 1.44 |
| Black | | 1.69 | 1.44 | 2.00 |  | 1.17 | 0.98 | 1.40 |
| Chinese | | 1.36 | 0.91 | 2.04 |  | 1.06 | 0.68 | 1.65 |
| Mixed | | 1.19 | 0.86 | 1.64 |  | 0.94 | 0.66 | 1.33 |
| Other | | 1.20 | 1.00 | 1.44 |  | 0.80 | 0.66 | 0.97 |
| Unknown | | 0.86 | 0.80 | 0.93 |  | 0.70 | 0.64 | 0.76 |
| **Socioeconomic deprivation quintile** | |  |  |  |  |  |  |  |
| 1 - Most deprived | | 0.81 | 0.76 | 0.87 |  | 0.78 | 0.72 | 0.84 |
| 2 | | 0.94 | 0.89 | 1.01 |  | 0.89 | 0.83 | 0.96 |
| 3 | | 0.88 | 0.83 | 0.93 |  | 0.87 | 0.82 | 0.93 |
| 4 | | 0.93 | 0.88 | 0.99 |  | 0.93 | 0.87 | 1.00 |
| 5 - Least deprived | | 1.00 | (reference) | |  | 1.00 | (reference) | |
| p-trend | | *<0.001* |  |  |  | *<0.001* |  |  |
| **Cancer Alliance** | |  |  |  |  |  |  |  |
| West Yorkshire and Harrogate | | 1.00 | (reference) | |  | 1.00 | (reference) | |
| Cheshire and Merseyside | | 0.09 | 0.07 | 0.10 |  | 0.07 | 0.06 | 0.09 |
| East Midlands | | 0.42 | 0.37 | 0.48 |  | 0.39 | 0.34 | 0.44 |
| East of England - North | | 0.23 | 0.20 | 0.27 |  | 0.22 | 0.19 | 0.26 |
| East of England - South | | 0.35 | 0.31 | 0.40 |  | 0.32 | 0.27 | 0.36 |
| Greater Manchester | | 0.10 | 0.08 | 0.11 |  | 0.08 | 0.07 | 0.10 |
| Humber, Coast and Vale | | 0.91 | 0.77 | 1.07 |  | 0.91 | 0.76 | 1.08 |
| Kent and Medway | | 0.17 | 0.15 | 0.21 |  | 0.15 | 0.13 | 0.18 |
| Lancashire and South Cumbria | | 0.20 | 0.17 | 0.24 |  | 0.19 | 0.16 | 0.22 |
| North Central and East London | | 0.59 | 0.51 | 0.68 |  | 0.53 | 0.45 | 0.62 |
| North East and Cumbria | | 0.21 | 0.18 | 0.24 |  | 0.19 | 0.16 | 0.22 |
| North West and South West London | | 0.29 | 0.25 | 0.33 |  | 0.24 | 0.20 | 0.28 |
| Peninsula | | 0.27 | 0.23 | 0.31 |  | 0.25 | 0.22 | 0.30 |
| Somerset, Wiltshire, Avon and Gloucestershire | | 0.24 | 0.21 | 0.28 |  | 0.22 | 0.19 | 0.26 |
| South East London | | 0.77 | 0.64 | 0.91 |  | 0.71 | 0.59 | 0.86 |
| South Yorkshire and Bassetlaw | | 0.89 | 0.75 | 1.05 |  | 0.89 | 0.74 | 1.06 |
| Surrey and Sussex | | 0.26 | 0.23 | 0.30 |  | 0.25 | 0.21 | 0.29 |
| Thames Valley | | 0.52 | 0.45 | 0.60 |  | 0.48 | 0.41 | 0.56 |
| Wessex | | 0.48 | 0.41 | 0.55 |  | 0.48 | 0.41 | 0.55 |
| West Midlands | | 0.31 | 0.28 | 0.35 |  | 0.29 | 0.26 | 0.33 |
| **Tumour characteristics** | |  |  |  |  |  |  |  |
| **Stage** | |  |  |  |  |  |  |  |
| I | | 1.00 | (reference) | |  |  |  |  |
| II | | 1.63 | 1.53 | 1.75 |  |  |  |  |
| III | | 1.66 | 1.55 | 1.77 |  |  |  |  |
| IV | | 1.04 | 0.97 | 1.12 |  |  |  |  |
| Unknown | | 0.58 | 0.54 | 0.62 |  |  |  |  |
| p-trend | | *<0.001* |  |  |  |  |  |  |
| **Grade** | |  |  |  |  |  |  |  |
| 1 | | 1.00 | (reference) | |  |  |  |  |
| 2 | | 2.29 | 2.03 | 2.58 |  |  |  |  |
| 3 | | 2.37 | 2.09 | 2.70 |  |  |  |  |
| 4 | | 0.78 | 0.37 | 1.53 |  |  |  |  |
| Unknown | | 0.38 | 0.34 | 0.44 |  |  |  |  |
| p-trend | | *<0.001* |  |  |  |  |  |  |
|  | OR = odds ratio, CI = confidence interval. ^a^ Univarable model ^b^ Multivariable model includes Cancer Alliance, age, gender, ethnicity and socioeconomic deprivation quintile | | | | | | | |
