# Supplementary material for: Identification of people with Lynch syndrome from those presenting with colorectal cancer in England: baseline analysis of the diagnostic pathway
Source: Eur J Hum Genet. 2024 Feb 15;32(5):529–38. doi: 10.1038/s41431-024-01550-w (PMC11061113; doi:10.1038/s41431-024-01550-w)
Supplement: Supplementary file 3 — Legends for Supplementary Material [file 41431_2024_1550_MOESM3_ESM.docx]

**671-23-EJHG: McRonald *et al*. Legends for Supplementary Material**

**Supplementary Figure 1:** Follow up molecular testing on CRCs diagnosed in 2019 that were identified as dMMR, split by Cancer Alliance. Follow up testing was defined as somatic *MLH1* promoter methylation or *BRAF* testing in MLH1 deficient / MSI-High CRCs, or germline MMR gene testing of patients with MSH2/MSH6/PMS2-deficient CRCs. Cancer Alliance is derived from patient postcode of residence at diagnosis, and based on 2019 boundaries. Note that there are some gaps in supply of *BRAF* data from the London and Thames Valley regions, so apparent testing rates for these areas may not be representative.

**Supplementary Table 1:** Likelihood of colorectal cancer patients receiving mismatch repair (MMR) testing through immunohistochemistry (IHC) or microsatellite instability (MSI) methods in England in 2019, according to patient and tumour characteristics and Cancer Alliance at diagnosis. Univariable and multivariable logistic regression modelling.
